# Influence of Tin Doped TiO_2_ Nanorods on Dye Sensitized Solar Cells

**DOI:** 10.3390/ma14216282

**Published:** 2021-10-21

**Authors:** Sandeep B. Wategaonkar, Vinayak G. Parale, Sawanta S. Mali, Chang-Kook Hong, Rani P. Pawar, Parvejha S. Maldar, Annasaheb V. Moholkar, Hyung-Ho Park, Balasaheb M. Sargar, Raghunath K. Mane

**Affiliations:** 1 Department of Chemistry, Sanjay Ghodawat Polytechnic, Atigre 416118, Maharashtra, India; sandip.wate@gmail.com; 2 DST-FIST Sponsored Material Research Laboratory, Department of Chemistry, Jaysingpur College, Shivaji University, Kolhapur 416001, Maharashtra, India; sargarbalasaheb@gmail.com; 3 Department of Chemistry, K. R. P. Kanya Mahavidyalaya, Shivaji University, Kolhapur 415409, Maharashtra, India; 4 Department of Materials Science and Engineering, Yonsei University, Seoul 03722, Korea; vinayakparale3@gmail.com; 5 Polymer Energy Materials Laboratory, School of Chemical Engineering, Chonnam National University, Gwangju 61186, Korea; sawanta@jnu.ac.kr (S.S.M.); hongck@jnu.ac.kr (C.-K.H.); 6 Department of Physics, Sanjay Ghodawat University, Kolhapur 416118, Maharashtra, India; rani.ddrrpp.pawar@gmail.com; 7 Department of Physics, D.Y. Patil College of Engineering, Salokhenagar, Kolhapur 416007, Maharashtra, India; parvezmaldar8@gmail.com; 8 Thin Films Nanomaterials Laboratory, Department of Physics, Shivaji University, Kolhapur 416004, Maharashtra, India; avmoholkar@gmail.com

**Keywords:** Sn-doped TiO_2_, Hydrothermal method, X-ray diffraction, photoelectrode, dye-sensitized solar cells

## Abstract

The one-step hydrothermal method was used to synthesize Sn-doped TiO_2_ (Sn-TiO_2_) thin films, in which the variation in Sn content ranged from 0 to 7-wt % and, further, its influence on the performance of a dye-sensitized solar cell (DSSC) photoanode was studied. The deposited samples were analyzed by X-ray diffraction (XRD) and Raman spectroscopy, which confirmed the existence of the rutile phase of the synthesized samples with crystallite size ranges in between 20.1 to 22.3 nm. In addition, the bare and Sn-TiO_2_ thin films showed nanorod morphology. A reduction in the optical band gap from 2.78 to 2.62 eV was observed with increasing Sn content. The X-ray photoelectron spectroscopy (XPS) analysis confirmed Sn^4+^ was successfully replaced at the Ti^4+^ site. The 3-wt % Sn-TiO_2_ based DSSC showed the optimum efficiency of 4.01%, which was superior to 0.87% of bare and other doping concentrations of Sn-TiO_2_ based DSSCs. The present work reflects Sn-TiO_2_ as an advancing material with excellent capabilities, which can be used in photovoltaic energy conversion devices.

## 1. Introduction

Nanostructured, nanoporous semiconducting metal oxides with large surface areas and high diffusion rates are exclusively utilized as photoanode materials in dye-sensitized solar cells (DSSCs) [1]. Due to their cost-effectiveness, ease of manufacturing, and higher light conversion efficiency, DSSCs have become important alternatives to traditional silicon solar cells [2,3]. Various metal oxide semiconductors, such as ZnO, TiO_2_, Nb_2_O_5_, and SnO_2_, have been explored as photoanode materials for the development of high-performance DSSCs [4,5,6,7]. The contributing factors that establish the TiO_2_ semiconductor electrodes as the best photoanodes are their charge transport capability and chemical stability. TiO_2_ plays a very important role in DSSCs as it provides a high surface for adsorption of dye, acquiring electrons from the excited dye state, and quickly transferring to fluorine-doped tin oxide (FTO). Since TiO_2_ possesses a large bandgap of about 3.2 eV, it cannot be efficiently used in the visible light region but is more sensitive to the ultraviolet region. Hence, different research groups have synthesized TiO_2_ by various methods to improve the edge of absorption, mostly in the visible region. Doping of different elements in TiO_2_ is one of the best ways to reduce the bandgap and change the electronic structure, as well as to minimize the recombination rate and promote visible light absorption. All the above parameters could help to improve the charge transfer rate, high open-circuit voltage (V_oc_), and will be beneficial to enhance the DSSCs’ efficiency [8,9].

Suitable metal ions doping in the TiO_2_ can cover up the grain boundaries, which will generate oxygen vacancies and can improve the photoelectrochemical properties of the photoanodes. Many metals are used for doping in the TiO_2_ host lattice, but Sn is the most influential because of the close ionic radius of Ti^4+^ = 0.60 Å and Sn^4+^ = 0.69 Å, which helps to suitably replace Ti^4+^ in TiO_2_ with Sn^4+^ ions to generate a homogenous mixture [10]. The Sn doping enhances the charge transport, photon absorption, and improves the surface quantum-dots loading density of TiO_2_, which results in high photoelectrochemical efficiency [11,12]. Xiang et al. prepared Ta added TiO_2_, which shifts the potential negatively and raises the concentration of electrons of the TiO_2_ electrode, resulting in the reduction of the electron recombination rate and electron transport by Ta doping [13]. Su et al. reported improvement in the short circuit current density (J_sc_) by Nb doping in TiO_2_. The enhancement in the J_sc_ is attributed to variation in the conduction band minimum (CBM), which facilitates electron transport and enhances the conductivity [14,15]. Different investigations have been done on doping TiO_2_ for the improvement of V_oc_ and J_sc_.

Different approaches have been used to synthesize Sn-doped TiO_2_ thin films which include spray pyrolysis [16,17], a microwave-assisted method [18], sol-gel [19,20,21], chemical co-precipitation [22], atomic layer deposition [23], a gas-phase oxidation method [24], and spin coating [25,26]. The Sn-doped TiO_2_ thin films have previously been used for different applications such as photocatalysis [27,28], gas sensing [29,30], lithium-ion batteries [31,32], and DSSCs [33,34]. Out of different synthesis processes available for the synthesis of TiO_2_ thin films, the hydrothermal method offers certain advantages such as the synthesis of rutile and anatase crystalline forms of TiO_2_ at relatively low temperatures, fine-tuning of morphology, and uniform coating onto the substrates [35].

In the present work, the hydrothermal method has been applied for the synthesis of Sn-doped TiO_2_ thin films using an acid catalyst. The Sn-TiO_2_ thin films are systematically synthesized by varying Sn concentration. The influence of Sn-doping concentration on structural, optical, morphological, and photoelectrical properties of Sn-TiO_2_ thin films has been examined. Improved photovoltaic performance is observed for the DSSCs fabricated with Sn-TiO_2_. Further, the Sn-doped TiO_2_ thin films have been successfully employed as photoelectrodes in DSSCs, which suggests a positive effect on its photovoltaic performance.

## 2. Methods

### 2.1. Synthesis of Sn-Doped TiO_2_ Films

Sn-doped TiO_2_ thin films were prepared by a single-step hydrothermal route by varying the Sn concentration in TiO_2_. FTO substrates were washed for 15 min by ultrasonic treatment using equal amounts of isopropyl alcohol, acetone, and deionized water (DIW) followed by drying in an N_2_ atmosphere. An equivalent volume of DIW and concentrated HCl was mixed and stirred for 10 min. In the aforementioned solution, 0.5 mL of titanium butoxide was mixed dropwise and constantly stirred for the next 30 min. The desired amount of tin (IV) chloride was poured into the aforesaid solution and stirred for the next 30 min until the solution becomes clear and homogeneous. The solution was then poured into the Teflon vessel and the ultrasonically cleaned FTO substrate was dipped in solution. The FTO substrate was kept inclined to the wall of the Teflon vessel with the conducting side facing upwards. The Teflon vessel was then fitted in the autoclave and heated for 3 h at 180 °C in a furnace. The autoclave was then allowed to cool naturally to room temperature. The FTO substrate coated with the desired material was then rinsed with DIW and dried at room temperature. The prepared films were finally annealed at 450 °C for 1 h. Sn-TiO_2_ thin films with different Sn concentrations (3, 5, and 7-wt %) were also prepared by the same procedure. Undoped TiO_2_ thin film was synthesized by the same procedure without the addition of the Sn precursor.

### 2.2. Cell Fabrication and Photovoltaic Measurements

Hydrothermally synthesized bare and Sn-doped TiO_2_ films with different concentrations of Sn were soaked in ethanolic 0.5 mM N719 dye (cisbis(isothiocyanato) bis (2,2′ bipyridyl-4,4′ dicarboxylato) ruthenium (II) bis-tetrabutylammonium, (Greatcell, Queanbeyan, Australia) solution for 24 h. After completion of the dye loading process, sensitized photoelectrodes were rinsed in acetonitrile and dried in air. DSSCs were fabricated by a two-electrode configuration comprising a working electrode and counter electrode. Dye-loaded bare TiO_2_ and Sn-TiO_2_ on FTO act as working electrodes, whereas platinum-coated FTO (Pt: FTO) acts as a counter electrode. Both these electrodes were sealed using polyacrylamide 1 mm spacers. The Pt: FTO electrodes were freshly prepared by drop-casting 0.5 mM hexachloroplatinic acid (H_2_PtCl_6_) dissolved in isopropanol solution onto FTO and heating at 400 °C for 20 min in air. The dye-loaded TiO_2_ photoanodes and platinum counter electrodes were closed with Surlyn thermoplastic (SX-1170-25, Solaronix, Aubonne, Switzerland) at specific temperatures and pressure. The electrolyte Iodolyte AN-50 was introduced through a pre-drilled Pt: FTO electrode opening. The system was eventually sealed with thermoplastics. The photovoltaic measurements were carried out at room temperature.

### 2.3. Characterizations

Rigaku Ultima X-ray diffractometer (Tokyo, Japan) with Cu K_α_ radiation, λ = 1.54 Å was used to investigate the structural characteristics. The WITec ALPHA 300 M Raman microscope was used to obtain the Raman spectra (excitation at 532 nm, 2.33 eV, Ulm, Germany). The chemical bonding of bare and doped TiO_2_ was examined using Fourier transform infrared spectroscopy (FTIR; Perkin Elmer 1760X spectrophotometer, Waltham, MA, USA) at wavelengths ranging from 400 cm^−1^ to 4000 cm^−1^. The surface morphologies were studied by using a field emission scanning electron microscope (FE-SEM, JSM-7001F, JEOL, Tokyo, Japan). The particle size was determined by using a JEM-2100F (JEOL, Tokyo, Japan) transmission electron microscope with an acceleration voltage of 125 kV. The Brunauer Emmett Teller (BET) N_2_ adsorption and desorption analyses were carried out by Quantachrome Instruments v10.0 (Florida, USA) to determine the surface area, pore size, and volume. The elemental composition of samples was analyzed using an X-ray photoelectron spectrometer (XPS, Thermo Scientific Inc., East Grinstead, UK) focused with Al (K_α_) monochromator (1486.6 eV) and the spot size was variable (30–400 µm in 5 µm steps). The absorbance spectra were obtained by Jasco spectrophotometer (Jasco V-770, Tokyo, Japan) with a wavelength ranging from 200 to 800 nm. The photovoltaic measurements were carried out with a solar simulator (McScience K201 LAB50, Suwon, Korea) for 10 s with AM 1.5 G with 1 sun (100 mW cm^−2^) illumination intensity. An NREL-calibrated Si solar cell with a KG-5 filter was used as a reference cell.

## 3. Results and Discussion

**Figure 1:**
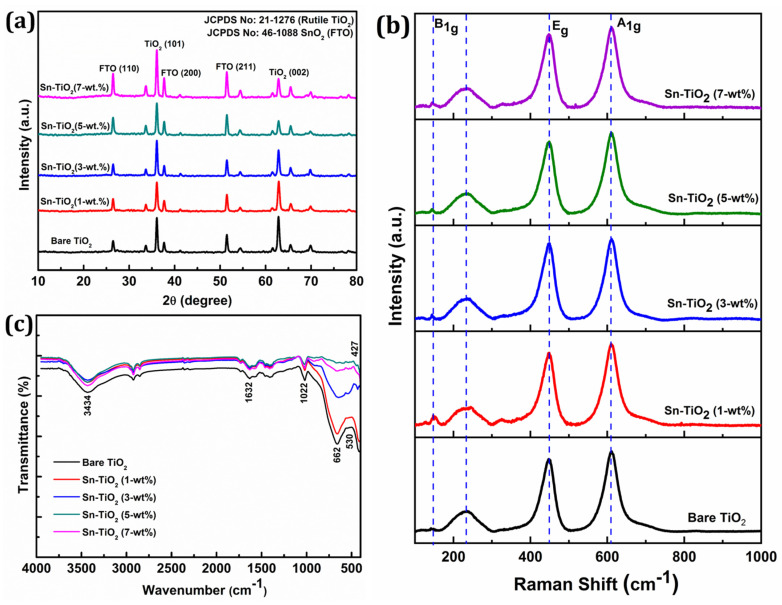
(**a**) XRD patterns, (**b**) Raman spectra, and (**c**) FT-IR spectra of hydrothermally synthesized bare TiO_2_, 1-wt %, 3-wt %, 5-wt %, and 7-wt % Sn-TiO_2_ thin films.

The X-ray diffraction (XRD) patterns of bare and Sn-TiO_2_ thin films are shown in Figure 1a. The Sn-TiO_2_ thin films have a tetragonal rutile crystal structure, which is the same as bare TiO_2_ (JCPDS No. 21-1276). Sn doping slightly affects the rutile TiO_2_ crystal structure. The (002) reflection of the Sn-TiO_2_ film is slightly displaced to a higher 2θ value and its intensity has decreased, as the concentration of Sn doping has increased from 1 to 7-wt %. The Sn doping has increased the intensity of (110) reflection. Due to Sn doping, only a slight variation in the lattice constants of bare-TiO_2_ thin films is observed, signifying the partial replacement of Ti^4+^ sites by Sn^4+^ cations due to their close ionic radii. The relevant crystal structure parameters, like the crystallite size, lattice constant, and cell volume, are shown in Table S1**.** The average crystallite size for the bare, 1, 3, 5, and 7-wt % Sn-TiO_2_ is determined as 20.1, 20.7, 21.2, 21.7, and 22.3 nm, respectively. This implies that with increasing Sn content, the average crystallite size of the samples increases. In all the samples, the rutile phase is the dominating phase, as shown in Figure 1a.

Figure 1b displays the Raman spectra of bare and Sn-TiO_2_ thin films. The prominent reflections positioned at 144, 448, and 610 cm^−1^ corresponding to the B1g, Eg, and A1g vibrational modes of the rutile phase, respectively. The intense reflections at 448 and 610 cm^−1^ represent the O–Ti–O bending and Ti–O stretching vibrations of the rutile phase, respectively [36]. The stretching vibration at 233 cm^−1^ can be assigned to compound vibration generated by a multi-phonon process, signifying the rutile phase of TiO_2_ [37]. The absence of characteristic reflections for SnO_2_ at 470 cm^−1^, 574 cm^−1^, 636 cm^−1^, and 776 cm^−1^ [38] confirms the non-existence of crystalline SnO_2_ due to minimal Sn doping concentration.

Figure 1c represents the FT-IR spectra of bare and Sn-TiO_2_ thin films in the 400–4000 cm^−1^ range. The bands located at 1600 and 3400 cm^−1^ can be assigned to the stretching and bending vibration of water molecules, respectively. In bare and Sn-TiO_2_, the bands at 1632 cm^−1^ represent the bending vibration of a water molecule and stretching vibration of O–H. The presence of a band over the range of 1022 to 1100 cm^−1^ indicates the evidence of Ti–O–Ti vibrations in the films [39]. Peaks observed in the 400–800 cm^−1^ range can be ascribed to vibration modes of Ti–O, Ti–O–Ti, Sn–O, and Ti–O–Sn bonds. The Sn doping into TiO_2_ results in a peak shift to a lower wavenumber observed in the FT-IR spectra of Sn-TiO_2_ [40]. The shifting of the peak from 662 to 651 cm^−1^ for Sn–TiO_2_ films confirms the Ti–O–Sn bond formation [18]. The band at 1460 cm^−1^ corresponds to the stretching vibration of the Ti–O–Ti, which has shifted to 1457 cm^−1^ because of Sn^4+^ doping [41]. The band observed at the low frequency region of FTIR spectra at 530 and 427 cm^−1^ corresponds to bending vibrations of Ti–O and Ti–O–Ti bonds [42].

**Figure 2:**
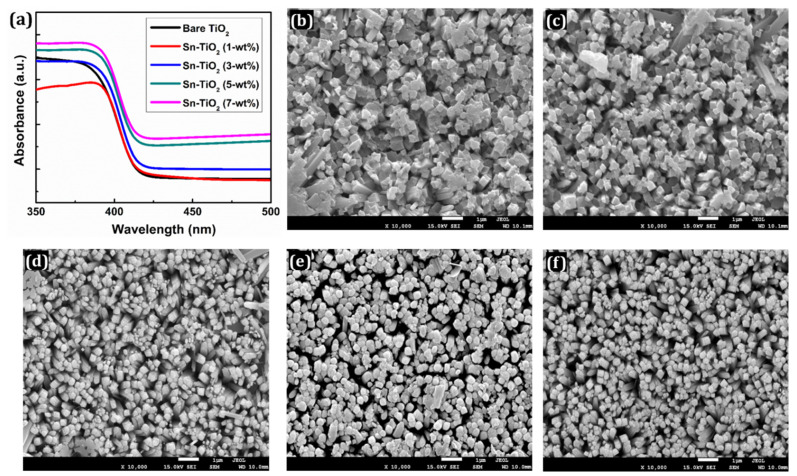
(**a**) UV-visible absorption spectra of bare TiO_2_, 1-wt %, 3-wt %, 5-wt %, and 7-wt % Sn-TiO_2_ thin films. FE-SEM images of (**b**) bare TiO_2_ (**c**) 1-wt %, (**d**) 3-wt %, (**e**) 5-wt %, and (**f**) 7-wt % Sn-TiO_2_ thin films.

Figure 2a presents the UV-Visible spectra of bare and Sn-TiO_2_ thin films prepared with different Sn doping concentrations. A small redshift is observed for the thin films and absorption band edges have broadened to a visible region with the rise in Sn concentration. This effect is mainly caused by Sn^4+^ doping, which generates a temporally indirect energy gap in the mid-gap zone of TiO_2_, facilitating an electron excitation and lowering the energy barrier needed for electrons to be excited from the valence band (VB) to the conduction band (CB). In comparison with bare TiO_2_, the absorption edge of Sn-doped TiO_2_ thin films has slightly shifted to a longer wavelength. The desired doping concentration thus increases the efficiency of cells in the visible region [43]. Figure S1 shows the Tauc plots of bare and Sn-TiO_2_ thin films, from which it is inferred that the bandgap has reduced from 2.78 to 2.62 eV with the increase in Sn content. The reduction in the bandgaps indicates that the minimum energy is required for the excitation of electrons. It facilitates the electron transfer from VB to CB.

In addition, the effect of Sn content on the surface morphology of the TiO_2_ thin films was determined using the FE-SEM technique as presented in Figure 2b–f. The FE-SEM images at a magnification of ×50,000 are shown in Figure S2a–e. The surface morphology appeared in the form of 1D nanorods for the bare TiO_2_, whereas for the Sn-TiO_2_ thin films, 3D flower-like nanorods are noticed, as shown in Figures S2a and S3a,b, respectively. The detailed growth mechanism of TiO_2_ nanorods has previously been explored [44]. The consistent growth of Sn-TiO_2_ nanorods is observed. These nanorods are mixed and assisted together to offer more interspace. Each nanorod is composed of a set of thinner nanorods with square top facets. These nanorods are nearly uniform, well-aligned, and uniformly distributed on the surface of the films. Nanorod morphology provides a larger surface area which enhances the efficiency of DSSCs by efficient transport of electrons. The whole surface of FTO is covered with well-aligned nanorods. The FTO substrate provides numerous nucleation centers which initiate the growth of the TiO_2_ nanorods. As shown in Figure 2b–f and Figure S2, the nanorods appeared less normal to the substrate, which can be attributed to the decreased intensity of (002) reflections of Sn-TiO_2_.

**Figure 3:**
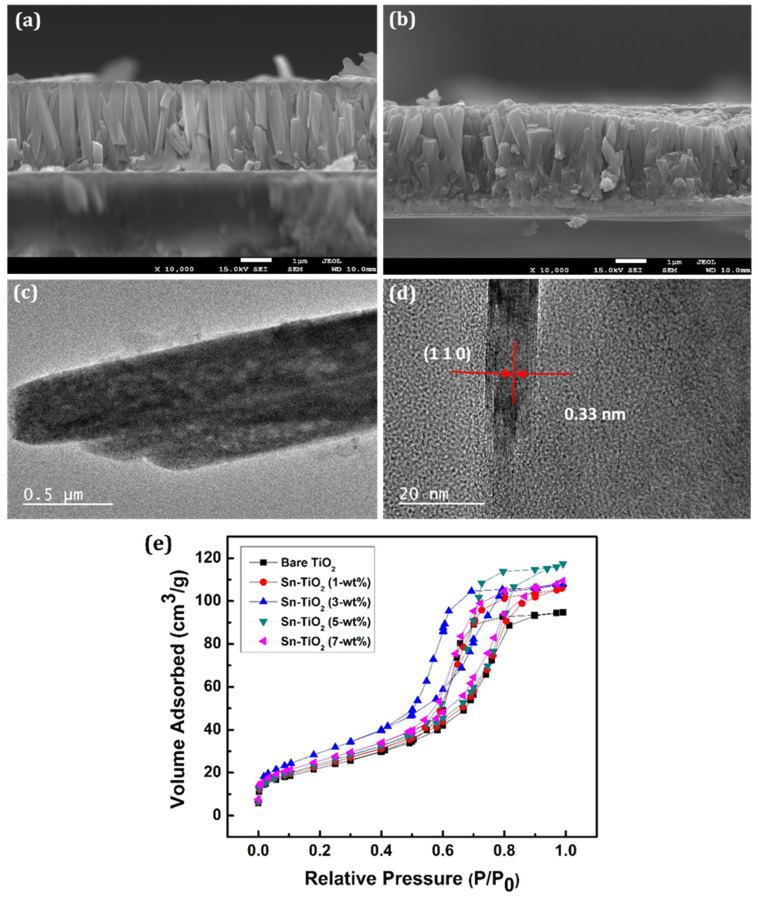
SEM cross-section of (**a**) 3-wt % (**b**) 7-wt % Sn-TiO_2_ thin films. HR-TEM image of 7-wt % Sn-TiO_2_ (**c**) Bundles of nanorods and (**d**) Lattice fringes with interplanar spacing of (110) reflection. (**e**) The N_2_ adsorption-desorption isotherms of the bare TiO_2_ and Sn-TiO_2_ thin films with varying Sn concentration.

Figure 3a,b shows the cross-section of 3-wt % Sn-TiO_2_ and 7-wt % Sn-TiO_2_ thin films, respectively, which indicates the formation of well-aligned nanorods. The cross-sectional view of 3-wt % and 7-wt % Sn-TiO_2_ thin films shows that the nanorods have grown almost perpendicular to the substrate. The average length and diameter of the nanorods are determined as 2.3 µm and 180 nm, respectively, for 3-wt % Sn-TiO_2_. For 7-wt % Sn-TiO_2_ thin films, the average length and diameter of the nanorods are estimated as 2.1 µm and 150 nm, respectively. The HR-TEM image of 7-wt % Sn-TiO_2_ thin films is depicted in Figure 3c. It reveals that numerous nanorods come together and form a bundle of nanorods. These nanorods are moderately uniform, with an average length of nearly 2 µm, and an average diameter of nearly 150 to 250 nm. Figure 3d displays the HR-TEM image of 7-wt % Sn-TiO_2_ thin films, in which lattice fringes of (110) reflection with interplanar spacing 0.33 nm are seen, which is a characteristic of rutile TiO_2_.

The BET analyses of TiO_2_ and Sn-TiO_2_ samples are carried out to investigate the changes in the surface area and pore diameter raised due to Sn doping. Figure 3e exhibits the BET N_2_ adsorption-desorption isotherms as representations of relative pressure (P/P_0_) against a volume of gas adsorbed at equilibrium. The presence of mesopores in the bare and Sn-TiO_2_ samples is confirmed by the presence of type IV isotherms with a significant hysteresis loop. The average specific surface areas of the polycrystalline bare-TiO_2_, 1, 3, 5, and 7-wt % Sn-TiO_2_ samples are determined as 80.69, 84.11, 107.57, 88.88, and 92.30 m^2^ g^−1^, respectively. The average pore volumes and mean pore diameters of bare-TiO_2_, 1, 3, 5, and 7-wt % Sn-TiO_2_ samples are calculated as 0.146, 0.165, 0.166, 0.181, and 0.169 cm^3^ g^−1^, and 7.25, 7.86, 6.20, 8.16, and 7.33 nm, respectively. It is observed that the surface area (S_BET_), pore-volume, and pore diameter of the bare and Sn-TiO_2_ thin films vary with Sn content. The 3-wt % Sn-TiO_2_ has the highest S_BET_ value, suitable for effective dye adsorption. The values of specific surface area, pore-volume, and mean pore diameter of bare-TiO_2_ and Sn-TiO_2_ samples have been illustrated in Table 1.

**Figure 4:**
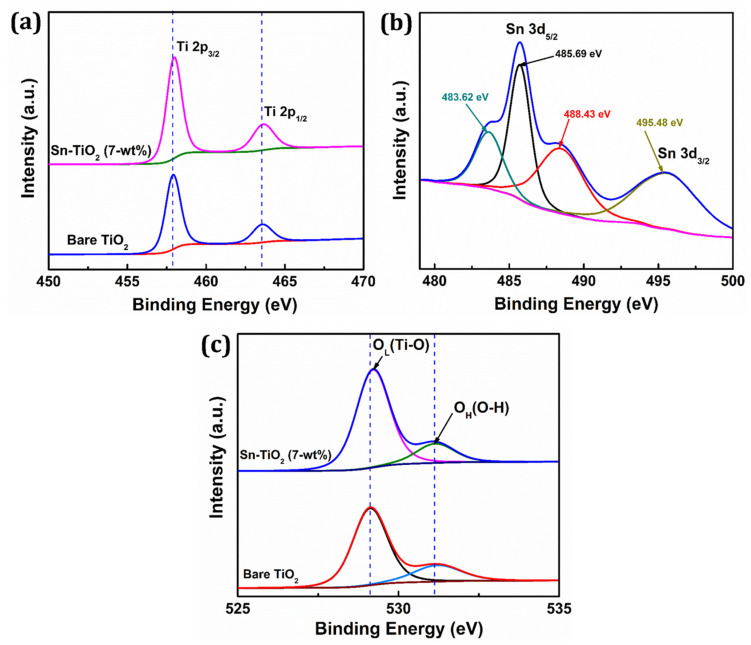
High-resolution XPS spectra of (**a**) Ti 2p, (**b**) Sn 3d, (**c**) O 1s fitting of bare TiO_2_ and 7-wt % Sn-TiO_2_ thin films.

The XPS technique is used to assess the chemical states and elemental composition of the prepared films. Figure S4 shows the survey spectrum of 7-wt % Sn-TiO_2_ thin film, which confirms the existence of titanium, tin, and oxygen elements in the synthesized film. The Sn 3d_3/2_ and Sn 3d_5/2_ peaks are detected for Sn-TiO_2_ thin films and are shown in the inset of Figure S4. Figure 4a displays the deconvoluted XPS spectra of Ti 2p of bare TiO_2_ and 7-wt % Sn-TiO_2_ thin films.

The binding energy for Ti 2p_3/2_ and Ti 2p_1/2_ for bare TiO_2_ is found to be 457.87 and 463.53 eV, respectively, whereas for Sn-TiO_2_, it is found to be 458.02 and 463.72 eV, respectively. When compared to the spectra of bare-TiO_2_, the two peaks of the Ti 2p spectra of 7-wt % Sn-TiO_2_ show a slight positive shift, which is most likely due to the interaction between Ti, O, and Sn atoms. It reveals that Sn doping induces a peak shift of Ti 2p to higher binding energy and indicates the +4 oxidation state of titanium [45]. This positive shift implies that electron loss from Ti^4+^ in the oxide is due to the existence of Sn^4+^ with considering their electronegativity difference (Ti = 1.54 and Sn = 1.96) [46]. The deconvoluted narrow scan XPS spectrum of Sn 3d is shown in Figure 4b. The binding energy 485.69 and 495.48 eV corresponds to Sn 3d_5/2_ and Sn 3d_3/2_ and confirms that Sn^4+^ is incorporated into the TiO_2_ lattice. Also, the deconvoluted Sn3d_5/2_ reflects two additional peaks at 483.62 and 488.43 eV, which are attributed to presence of Sn^2+^ and Sn^4+^ oxidation states, respectively [47,48]. Figure 4c represents the deconvoluted O 1s spectra of bare TiO_2_ and 7-wt % Sn-TiO_2_ thin films. The intense peaks observed at 529.10 eV and 529.23 eV are due to lattice oxygen (Ti–O) and the less intense peaks at 531.14 eV and 531.18 eV correspond to adsorbed hydroxyl (–OH) group in the bare-TiO_2_ and Sn-TiO_2_ thin films, respectively. The peak shifting to higher binding energy due to Sn doping is observed for Sn-TiO_2_ thin films. These findings support the existence of the Ti–O–Sn bond in Sn-doped films because of the replacement of Ti^4+^ by Sn^4+^.

**Figure 5:**
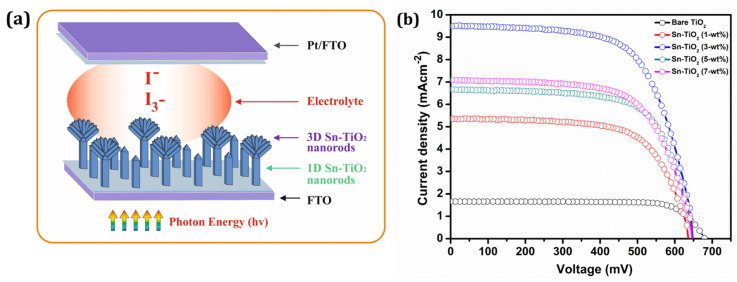
(**a**) The graphical view of 1D and 3D Sn-TiO_2_ nanorods based DSSCs (**b**) The Photocurrent density–photovoltage (J–V) curves of dye-sensitized bare TiO_2_ and Sn-TiO_2_ photoelectrodes with different Sn concentrations.

The graphical view of 1D and 3D Sn-TiO_2_ nanorods for the application of DSSCs is depicted in Figure 5a. The 3D Sn-TiO_2_ flower-like morphology comprises single-crystalline nanorods, which enhance electron mobility and photon harvesting through light scattering (Figure S5). Furthermore, the flower-like morphology provides a higher surface area for dye loading, and thus more absorption takes place towards the visible region.

Figure 5b represents the J–V curves of the DSSCs constructed using bare-TiO_2_ and 1, 3, 5, and 7-wt % Sn-TiO_2_ thin films sensitized with ruthenium dye N719. Table 2 summarizes the different DSSCs performance parameters of bare-TiO_2_ and 1, 3, 5, and 7-wt % Sn-TiO_2_ thin films. The cell configurations employed to measure J–V characteristics were FTO/TiO_2_/N719 Dye/I^-^-I_3_^-^/Pt: FTO and FTO/Sn-TiO_2_/N719 dye/I^-^-I_3_^-^/Pt: FTO.

The magnitude of the J_sc_ observed is 1.65 mA cm^−2^ for the bare-TiO_2_ and 5.35, 9.49, 6.66, and 7.08 mA cm^−2^ for 1, 3, 5, and 7-wt % Sn-TiO_2_ thin films, respectively. The DSSCs with bare TiO_2_ thin films provided power conversion efficiency (PCE) of 0.87% with a fill factor (FF) of 77.70%. It is observed that the variation in the J_sc_ values is governed by variation in the Sn concentration. As compared to bare TiO_2_, the enhancement in the PCE of Sn-TiO_2_ based DSSCs is observed. The possible reason for the enhancement in the PCE of Sn-TiO_2_ is ascribed to the large surface area of vertically grown nanorods that may enhance the output photocurrent. Moreover, the morphology observed for 3-wt % Sn-TiO_2_ thin films is a uniformly distributed, well-aligned bundle of nanorods (Figure S3a), which offers a greater surface area for dye loading and facilitates the transport of electrons. For the DSSCs fabricated with 3-wt % Sn-TiO_2_ thin films, the highest J_sc_ of 9.49 mA cm^−2^ is observed with V_oc_ of 0.64 V, and it has provided the highest PCE of 4.01%.

The Sn-TiO_2_ nanorods are found to be capable of enhancing the efficiency of DSSCs by providing a confined path for the transport of charge carriers with redox electrolyte and rapid charge separation. The PCE of Sn-TiO_2_ photoelectrode is marginally higher than bare TiO_2_ photoelectrodes due to a modest rise in defect states, leading to less recombination in the trapped sites. The interaction of Sn with the TiO_2_ lattice generates a shift in the Fermi level of TiO_2_. Consequently, the Sn captures the electron within the conduction band of TiO_2_. In the meantime, the equilibrium of the Fermi level is achieved [49]. Electron capture by Sn extends the lifespan of holes and reduces hole–electron recombination. Hence, noticeable improvement in the J_sc_ and V_oc_ is observed. Doped photoelectrode cells have a much higher performance than pure photoelectrode cells due to enhanced chemisorptions onto doped TiO_2_ [50]. The substitution of Sn causes distortion in the TiO_2_ lattice, creates oxygen vacancies, and contributes to enhanced photo activity. The improvement of the Sn-doped photoelectrode-based cell implies that doping may generate a donor level, leading to a greater concentration of the carrier with a decrease in film resistance [51]. For ideal DSSCs, the R_s_ (series resistance) should be low, and R_sh_ (shunt resistance) should be a maximum. The current-voltage characteristics depends upon R_s_ and R_sh_ resistance. A lower R_s_ enhances current flow in the circuit, and high R_sh_ provides fewer shorts or leaks in the device. In an ideal cell, the R_s_ value is close to zero, and R_sh_ approaches infinity. The R_s_ and R_sh_ are measured by the J–V curve and can be determined by Equations (1) and (2).

(1) $${\left(\frac{\mathrm{dI}}{\mathrm{dV}}\right)}_{I=o}=\left(\frac{1}{R_{s}}\right)$$

(2) $${\left(\frac{\mathrm{dI}}{\mathrm{dV}}\right)}_{V=o}=\left(\frac{1}{R_{\mathrm{sh}}}\right)$$

The bare-TiO_2_ showed the lowest efficiency with a high R_s_ value of 168.66 Ω, whereas the 3-wt % Sn-TiO_2_ films exhibited higher efficiency with a low R_s_ value of 56.2 Ω. These findings confirmed that low R_s_ and high R_sh_ determine the PCE of the DSSCs.

The tin doping reduces the band gap of TiO_2_. The charge transfer from the bulk to the surface of nanorods reduces the bandgap and facilitates the photogeneration process. The concentration of impurities increases with dopant concentration. Such impurities act as charge trapping sites for hole–electron recombination. If a doping content is more or less than 3-wt %, the recombination rate is more severe and results in a reduction in V_oc_ and J_sc_ and thus affects the PCE. Therefore, 3-wt % Sn-TiO_2_ thin films showed the optimum quantity of dopant, which gives better PCE.

## 4. Conclusions

We have designed Sn-TiO_2_ nanorod morphology-based DSSCs using a simple, low temperature, and low-cost hydrothermal method. The synthesized bare and Sn-TiO_2_ thin films with nanorod morphology have been successfully sensitized with N719 dye using the piranha solution etching process. The Sn-doped TiO_2_ facilitates the rutile phase formation at a certain annealing temperature and reduces the band gap of TiO_2_. The 3-wt % Sn-TiO_2_ showed a specifically large surface area of about 107.57 m^2^ g^−1^ which adsorbs more dye and thereby improves the performance of DSSCs. Photovoltaic studies showed considerable enhancement in PCE for the 3-wt % sample with a maximum J_sc_ of 9.49 mA cm^−2^ with a V_oc_ of 0.64 V and a PCE of 4.01%. Hence, Sn-TiO_2_ photoelectrodes will be promising photoelectrode materials for the development of highly efficient DSSCs.

## Figures and Tables

**Table 1:** Surface area, pore volume, and pore diameters of bare-TiO_2_ and Sn-TiO_2_ thin films.

Samples	Surface Area (m^2^ g^−1^)	Total Pore Volume (cm^3^ g^−1^)	Mean Pore Diameter (nm)
Bare TiO_2_	80.69	0.146	7.25
Sn-TiO_2_ (1-wt %)	84.11	0.165	7.86
Sn-TiO_2_ (3-wt %)	107.57	0.166	6.20
Sn-TiO_2_ (5-wt %)	88.88	0.181	8.16
Sn-TiO_2_ (7-wt %)	92.30	0.169	7.33

**Table 2:** Performance characteristics of DSSCs based on the bare-TiO_2_ and Sn-TiO_2_ photoelectrodes with varying Sn concentrations.

DSSCs	J_sc_ (mA cm^−2^)	V_oc_ (V)	FF (%)	R_sh_ Ω	R_s_ Ω	η (%)
Bare TiO_2_	1.65	0.68	77.70	355881	168.6	0.87
Sn-TiO_2_ (1-wt %)	5.35	0.63	66.43	11760	86.6	2.26
Sn-TiO_2_ (3-wt %)	9.49	0.64	65.29	8769	56.2	4.01
Sn-TiO_2_ (5-wt %)	6.66	0.64	68.17	12518	57.4	2.93
Sn-TiO_2_ (7-wt %)	7.08	0.64	65.64	10212	56.6	3.00

## Data Availability

Inside the article, data is included.

## Supplementary Materials

The following are available online at https://www.mdpi.com/article/10.3390/ma14216282/s1, Figure S1: Tauc plots showing bandgap of synthesized thin films having a different concentration of Sn; Figure S2: FE-SEM images of (a) bare TiO_2_, (b) 1-wt %, (c) 3-wt %, (d) 5-wt % and (e) 7-wt % Sn-TiO_2_ thin films with magnification of ×50,000; Figure S3: FE-SEM images of (a) 3-wt % Sn-TiO_2_ and (b) 7-wt % Sn-TiO_2_ showing epitaxial growth of nanorods forming flower-like morphology; Figure S4: XPS survey spectrum of 7-wt % Sn-TiO_2_; Figure S5: Morphological variation from nanorods to nano-flower; Table S1: Crystal structure parameters of bare-TiO_2_ and Sn-TiO_2_ thin films.
