# Supplementary material for: Influence of Tin Doped TiO2 Nanorods on Dye Sensitized Solar Cells
Source: Materials (Basel). 2021 Oct 21;14(21):6282. doi: 10.3390/ma14216282 (PMC8585088; doi:10.3390/ma14216282)
Supplement: Supplementary file 1 [file materials-14-06282-s001.zip › materials-1416221-supplementary.pdf]

materials

MDPI

Supplementary Materials

Influence of Tin Doped TiO_2 Nanorods on Dye Sensitized Solar Cells

Sandeep B. Wategaonkar [1,2,3], Vinayak G. Parale [4], Sawanta S. Mali [5], Chang-Kook Hong [5], Rani P. Pawar [6], Parvejha S. Maldar [7,8], Annasaheb V. Moholkar [8], Hyung-Ho Park [4,*], Balasaheb M. Sargar [2] and Raghunath K. Mane [3,*]

[1] Department of Chemistry, Sanjay Ghodawat Polytechnic, Atigre, 416118, Maharashtra, India; sandip.wate@gmail.com
[2] Department of Chemistry, DST-FIST Sponsored Material Research Laboratory, Jaysingpur College (Affiliated to Shivaji University, Kolhapur), Jaysingpur 416001, Maharashtra, India; sargarbalasaheb@gmail.com
[3] Department of Chemistry, K. R. P. Kanya Mahavidyalaya (Affiliated to Shivaji University, Kolhapur), Uran-Islampur 415409, Maharashtra, India
[4] Department of Materials Science and Engineering, Yonsei University, Seoul 03722, Korea; vinayakparale3@gmail.com (V.G.P.); hhpark@yonsei.ac.kr (H.-H.P.)
[5] Polymer Energy Materials Laboratory, School of Chemical Engineering, Chonnam National University, Gwangju 61186, Korea; sawanta@jnu.ac.kr (S.S.M.); hongck@jnu.ac.kr (C.-K.H.)
[6] Department of Physics, Sanjay Ghodawat University, Kolhapur 416118, Maharashtra, India; rani.ddrrpp.pawar@gmail.com
[7] Department of Physics, D.Y. Patil College of Engineering, Salokhenagar, Kolhapur 416007, Maharashtra, India; parvezmaldar8@gmail.com
[8] Thin Films Nanomaterials Laboratory, Department of Physics, Shivaji University, Kolhapur 416004, Maharashtra, India; avmoholkar@gmail.com
* Correspondence: rkmanekrp1970@gmail.com (R.K.M.); hhpark@yonsei.ac.kr (H.-H.P.); Tel.: +91-992-148-2155 (R.K.M.); +82-2-2123-2853 (H.-H.P.)

Citation: Wategaonkar, S.B.; Parale, V.G.; Mali, S.S.; Hong, C.-K.; Pawar, R.P.; Maldar, P.S.; Moholkar, A.V.; Park, H.-H.; Sargar, B.M.; Mane, R.K. Influence of Tin Doped TiO_2 Nanorods on Dye Sensitized Solar Cells. *Materials* **2021**, *14*, 6282. https://doi.org/10.3390/ma14216282

Academic Editor: Vincenzo Baglio

Received: 26 September 2021
Accepted: 17 October 2021
Published: 21 October 2021

Publisher's Note: MDPI stays neutral with regard to jurisdictional claims in published maps and institutional affiliations.

Copyright: © 2021 by the authors. Licensee MDPI, Basel, Switzerland. This article is an open access article distributed under the terms and conditions of the Creative Commons Attribution (CC BY) license (http://creativecommons.org/licenses/by/4.0/).

Materials:

Titanium butoxide ($Ti{\cdot}[O{\cdot}(CH_2)_3CH_3]_4$, 97%), Substrate Fluorine doped tin oxide (FTO) (F: SnO_2, ~8 Ω/cm, 2.5 cm × 2.5 cm), Tin (IV) Chloride ($SnCl_4{\cdot}5H_2O$) were obtained from Sigma Aldrich (St. Louis, Mo. USA). Nitric acid (HNO_3, 38%), sulfuric acid (H_2SO_4, 99.9%), and hydrochloric acid (HCl, 35.40%), were purchased from Thomas Baker (Mumbai, India). Absolute ethanol (99.9%), Isopropyl alcohol (99.9%) were obtained from Changshu Hongsheng fine chemicals (Changshu, China) and Sisco research laboratory (New Mumbai, India), respectively. The N719 dye (Di-Tetrabutylammonium cis-bis (isothiocyanato) bis (2, 2-bipyridyl-4, 4′-dicarboxylato)-ruthenium (II), 95% (NMR)) obtained from Greatcell (Queanbeyan, Australia) was employed as sensitizer in the present work. Redox electrolyte Iodolyte AN-50 (Solaronix, Aubonne, Switzerland) was applied in DSSCs. All the chemicals were utilized as received.

Table S1. Crystal structure parameters of bare-TiO_2 and Sn-TiO_2 thin films.

Sample	Lattice constant a (Å)	Lattice constant c (Å)	Crystallite size (nm)	Cell Volume (Å^3)
Bare TiO_2	4.69	2.94	20.1	64.66
Sn-TiO_2 (1-wt %)	4.71	2.95	20.7	65.44
Sn-TiO_2 (3-wt %)	4.73	2.96	21.2	66.22
Sn-TiO_2 (5-wt %)	4.70	2.94	21.7	64.94
Sn-TiO_2 (7-wt %)	4.72	2.96	22.3	65.94

Equation (1) can be used to calculate the lattice parameters a and c.

$$\frac{1}{d^2} = \frac{h^2 + k^2}{a^2} + \frac{l^2}{c^2} \quad (1)$$

Where d is the interplanar spacing and (hkl) are the Miller indices, respectively.

Equation (2) represents the Debye-Scherrer's equation and can be used to calculate the average crystallite size.

$$D = \frac{0.9\lambda}{\beta\cos\theta} \quad (2)$$

Where D = crystallite size, β = full-width half maximum, λ= wavelength, and θ = Bragg's angle of diffraction.

Optical Studies:

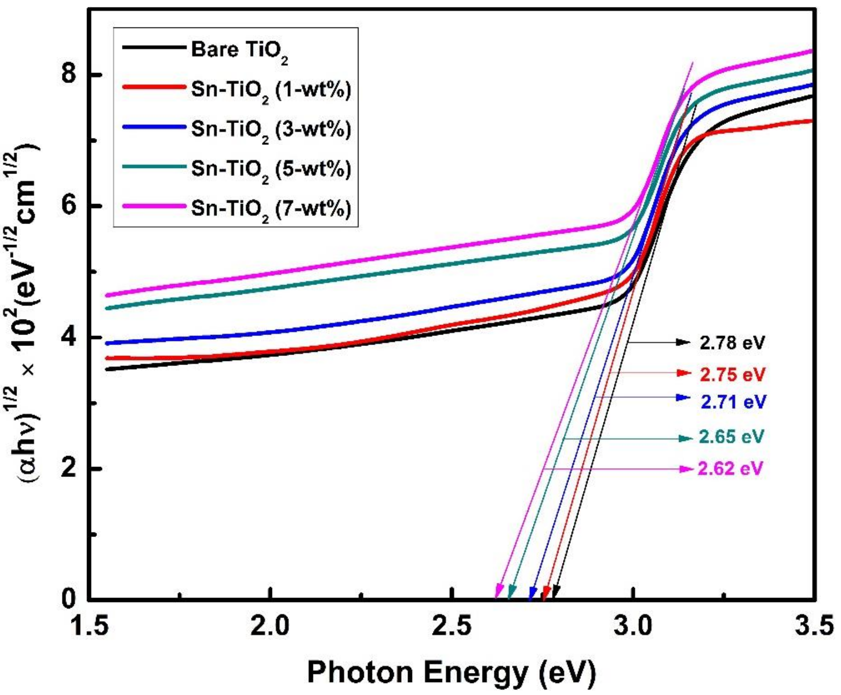

Figure S1. Tauc plots showing the band gap of synthesized thin films having a different concentration of Sn.

The band gap can be determined using the Equation (3).

$$\boldsymbol{\alpha = \frac{A(h\upsilon - Eg)^n}{h\upsilon}} \quad (3)$$

Where, α, Eg and hυ are the coefficient of absorption, optical band gap, and photon energy respectively. 'A' is an energy-independent constant, and 'n' is an optical transition constant. The optical band gap is calculated using a Tauc plot by concluding the straight portion of $(\alpha h\nu)^{1/2}$ against photon energy (hν) presented in Fig S1. TiO_2 is an indirect band gap semiconductor that shows the indirect and allowed transition.

FE-SEM:

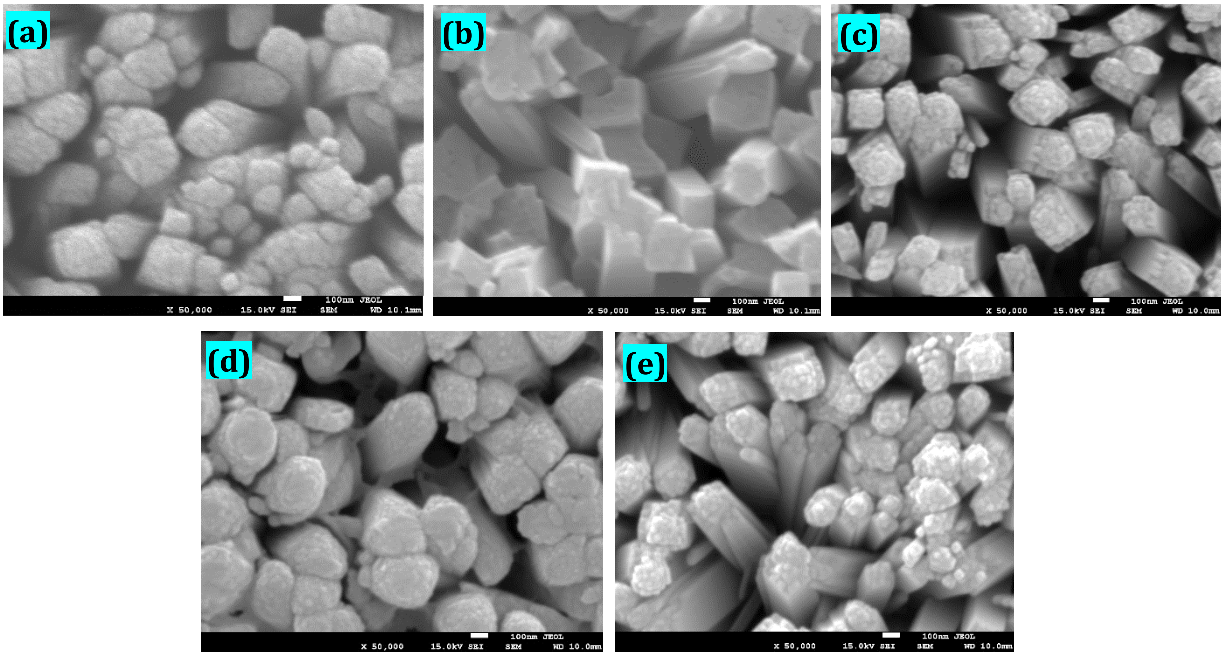

Figure S2. FE-SEM images of (**a**) bare TiO_2, (**b**) 1-wt %, (**c**) 3-wt %, (**d**) 5-wt % and (**e**) 7-wt % Sn-TiO_2 thin films with magnification of ×50,000.

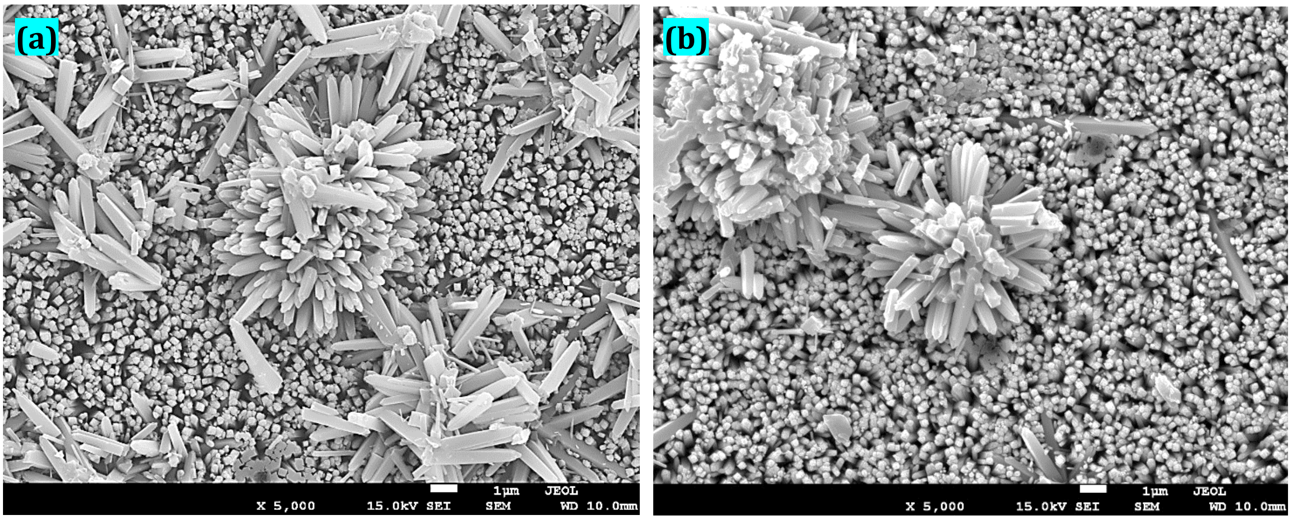

Figure S3. FE-SEM images of (**a**) 3-wt % Sn-TiO_2 and (**b**) 7-wt % Sn-TiO_2 showing epitaxial growth of nanorods forming flower-like morphology.

XPS:

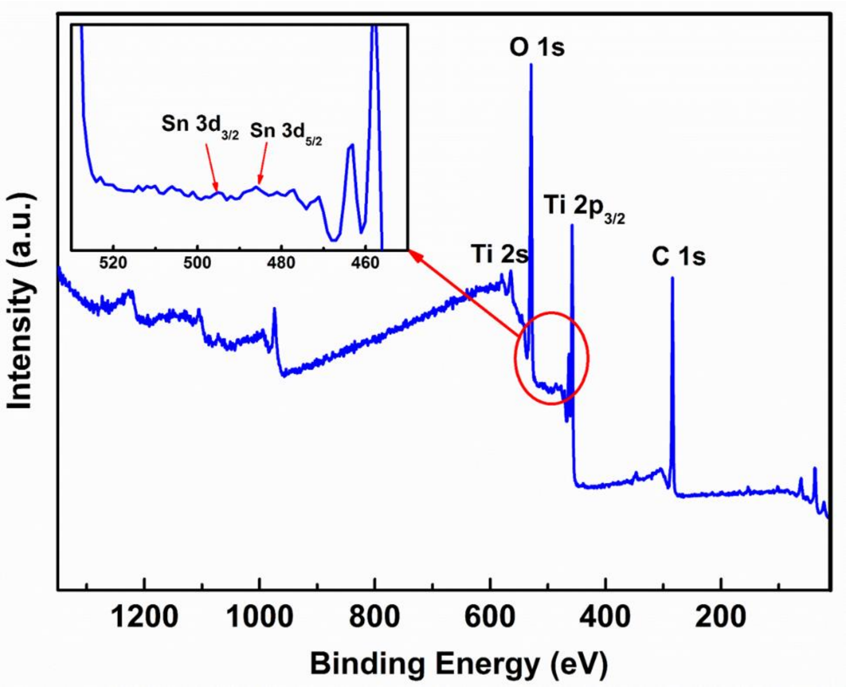

Figure S4. XPS survey spectrum of 7-wt % Sn-TiO_2.

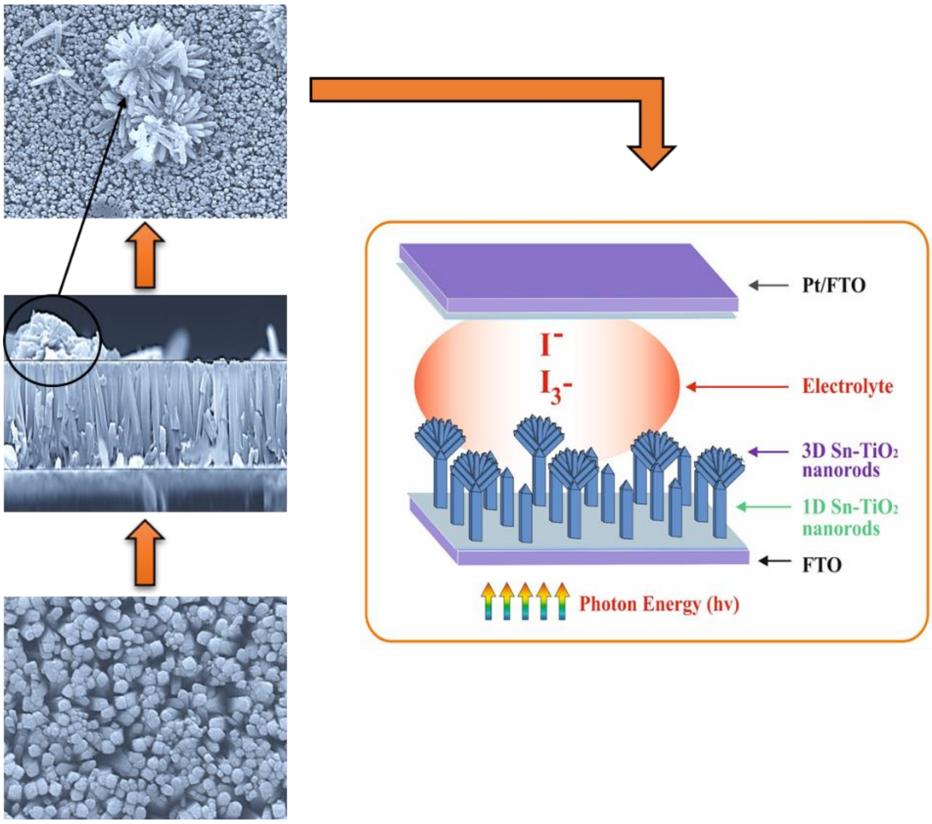

Figure S5. Morphological variation from nanorods to nano-flower

J-V Characteristics:

The power conversion efficiency (PCE) (η) of the DSSCs can be calculated using Equation (4):

$$\eta = \frac{J_{sc}V_{oc}}{P_{in}} \times FF \times 100 \quad (4)$$

where J_{sc} = short circuit current density, V_{oc} = open-circuit voltage, FF = fill factor, and P_{in} = power density.

The Fill factor (FF) can be calculated using Equation (5):

$$FF = \frac{J_{max} \times V_{max}}{J_{sc} \times V_{oc}} \quad (5)$$

where J_{max} = maximum current density and the V_{max} = maximum voltage.
